# Transcriptomic profiling of pemphigus lesion infiltrating mononuclear cells reveals a distinct local immune microenvironment and novel lncRNA regulators

**DOI:** 10.1186/s12967-022-03387-7

**Published:** 2022-04-21

**Authors:** Zi-xuan Huang, Peng Qu, Kan-kan Wang, Jie Zheng, Meng Pan, Hai-qin Zhu

**Affiliations:** 1 Department of Dermatology, Rui Jin Hospital Affiliated to Shanghai Jiao Tong University School of Medicine, Shanghai, 200025 China; 2 Shanghai Institute of Hematology, State Key Laboratory of Medical Genomics, National Research Center for Translational Medicine at Shanghai, Ruijin Hospital Affiliated to Shanghai Jiao Tong University School of Medicine, Shanghai, 200025 China

**Keywords:** Pemphigus, Skin immune infiltrates, Microarray, lncRNA, Biomarker

## Abstract

Pemphigus is an autoimmune skin disease. Ectopic lymphoid-like structures (ELSs) were found to be commonly present in the pemphigus lesions, presumably supporting in situ desmoglein (Dsg)-specific antibody production. Yet functional phenotypes and the regulators of Lymphoid aggregates in pemphigus lesions remain largely unknown. Herein, we used microarray technology to profile the gene expression in skin lesion infiltrating mononuclear cells (SIMC) from pemphigus patients. On top of that, we compared SIMC dataset to peripheral blood mononuclear cells (PBMC) dataset to characterize the unique role of SIMC. Functional enrichment results showed that mononuclear cells in skin lesions and peripheral blood both had over-represented IL-17 signaling pathways while neither was characterized by an activation of type I Interferon signaling pathways. Cell-type identification with relative subsets of known RNA transcripts (CIBERSORT) results showed that naïve natural killer cells (NK cells) were significantly more abundant in pemphigus lesions, and their relative abundance positively correlated with B cells abundance. Meanwhile, plasma cells population highly correlated with type 1 macrophages (M1) abundance. In addition, we also identified a lncRNA LINC01588 which might epigenetically regulate T helper 17 cells (Th17)/regulatory T cells (Treg) balance via the peroxisome proliferator-activated receptor (PPAR) signaling pathway. Here, we provide the first transcriptomic characterization of lesion infiltrating immune cells which illustrates a distinct interplay network between adaptive and innate immune cells. It helps discover new regulators of local immune response, which potentially will provide a novel path forward to further uncover pemphigus pathological mechanisms and develop targeted therapy.

**Supplementary Information:**

The online version contains supplementary material available at 10.1186/s12967-022-03387-7.

## Background

Pemphigus is a group of life-threatening autoimmune diseases characterized by intraepidermal blistering and autoantibodies against epidermal structural proteins such as Dsg 1 and Dsg 3 [1]. Topical use of corticosteroids alone has shown promising results in some pemphigus patients [2]. The underlying mechanism of effective topical corticosteroids is currently unknown. Our previous research has provided important insights: abundant infiltrating T cells and Ig + B cells have been found in pemphigus lesions [3–5]. These T-B cells took part in forming ELSs, a structure which is conducive to antibodies secretion, ranging from tight clusters of T-B cells to highly organized structures that comprise functional germinal centers [5]. We have established that skin infiltrating lymphocytes in pemphigus lesions can produce Dsg1/3 antibodies in vitro which makes them valuable study subjects. An enhanced understanding of the genetic basis of these largely unexplored immune cells is a requisite to advance the search for a more targeted therapy.

The use of transcriptome analysis has been a key method in uncovering the latent mechanism that may be causing or compounding diseases. Microarray expression profiling of human PBMC has identified novel therapeutic targets and promising diagnostic biomarkers for autoimmune diseases [6–9]. However, as skin harbors a pool of innate and adaptive immune cells constituting a complex network, studies of peripheral blood may not reflect the local immune responses in skin lesions. By B cell receptor repertoire sequencing, we have previously revealed that certain clones of lesional B cells expanded locally in pemphigus [5]. Hence, we aim to further characterize the compositions and dynamics of immune infiltrates in lesions. Meanwhile, increasing evidence has shown that immune responses are not only regulated by signaling pathways but also by epigenetic mechanisms involving DNA methylation, histone modification and non-coding RNAs (ncRNA) [10]. Changes of lncRNAs (ncRNA transcripts > 200 bp) are especially pervasive in human autoimmune diseases [11]. lncRNAs possess various biological functions, such as regulating protein and RNA stability as well as protein-DNA interaction. Yet, little is known about lncRNA expression profile in pemphigus. As a valuable model of organ-specific humoral autoimmune disease, transcriptome analysis of pemphigus, including lncRNA and mRNA, may help to identify novel autoimmunity-promoting genes.

In this study, both SIMC and PBMC microarray datasets were analyzed. We first screened out DEGs between pemphigus and healthy samples, then compared two sample sources (peripheral blood and lesions) to uncover their transcriptomic difference. CIBERSORT and GSEA were used to evaluate the abundance of immune cells and analyze the mechanism by which those immune infiltrates may affect pemphigus pathogenesis. Subsequently, both datasets were integrated and analyzed by WGCNA and cystoscope in attempt to discover pathogenesis related modules. Our findings corroborate the involvement of local immune dysregulation and altered Immune cell composition as potential drivers of pemphigus lesions. Moreover, we constructed a lncRNA-mediated competing endogenous RNA (ceRNA) network and identified epigenetic regulators, such as LINC01588 which might modulate Treg/Th17 balance via PPAR signaling pathway. Our study shed lights on the microenvironment at skin lesions and its potential epigenetic regulatory mechanism in pemphigus.

## Method

### Patient recruitment and ethical approval

Skin biopsies were collected from 4 patients with pemphigus, and 4 from age- and sex-matched healthy donors. In the pemphigus group, only blisters or erosions skin lesions were collected. Blood samples were also collected from 4 patients with pemphigus, and 4 from age- and sex-matched healthy donors. All the patients were diagnosed with pemphigus foliaceus or pemphigus vulgaris and had not been treated with systemic therapy before the study. The diagnoses were confirmed with clinical manifestations, histology, Dsg-specific antibody tests and immunohistology criteria. Shanghai Jiao Tong University School of the Medicine Research Ethics Committee approved the study. Written informed consent was obtained from all subjects before involving them in the study.

### Sample collection, skin cell preparation, and mononuclear cell preparation

1cm^2^ sized skin biopsy samples from four patients with pemphigus and four healthy donors were collected and incubated in a buffer containing collagenase IV, hyaluronidase, and DNase-I (Sigma-Aldrich, St. Louis, MO) for digestion at 37 °C for 2 h. After digestion, the samples were passed through a 70 mm cell strainer (BD Biosciences, USA), and single cell suspensions were obtained. Mononuclear cells were isolated from skin tissue single cell suspensions by density separation gradient using Lymphoprep solution (Axis-shield, Norway) and resuspended in RPMI 1640 (Invitrogen, USA) medium supplemented with 1 ml 5% fetal bovine serum (FBS; Sigma-Aldrich, USA) after washed with phosphate buffer saline. 4 ml blood samples were collected from a total of 8 participants (4 pemphigus patients and 4 healthy controls), from which PBMCs were isolated by density separation gradient using Lymphoprep (Stemcell Technologies, Vancouver, Canada) within 4 h since blood collection.

### RNA extraction, quality, and integrity determination

Ranging from 3.0*10^5^ to 8*10^5^ cells, Lymphoprep isolated mononuclear cells derived from each sample were prepared for further experimentation. Total RNA was extracted from the mononuclear cells of a pemphigus lesion and normal skin using Trizol (Invitrogen, USA). Purity and concentration of isolated total RNA were measured using a NanoDrop® UV–Vis spectrophotometer (Thermo Fisher, USA). Sampling and RNA isolation was performed by the same personnel using the same methodology.

### Transcriptome microarray

The isolated RNA was labeled and hybridized on an Arraystar Human LncRNA Microarray v4.0 (Arraystar, USA) according to instructions on the expression manual by the KangChen Bio-tech Corporation (Shanghai, China). The Arraystar microarray detects a comprehensive collection of 40,173 LncRNAs including 7506 well annotated LncRNAs (Gold Standard) and 32,667 high confidence LncRNAs (Reliable) constructed using highly reputable public transcriptome database (Refseq, UCSC knowngenes, Ensembl). The array also includes a collection of 20,730 protein coding mRNAs supported by Universal Protein Resource (Uniprot) database. Further bioinformatic analysis was carried out in a R environment. Transcriptome data of PBMC was acquired from our previous study [12].

### Differential expression analysis and functional enrichment

Raw signal intensity was converted into normalized and summarized expression data which was used as input for the linear models for microarray data analysis algorithm (LIMMA) to assess differential expression of genes between pemphigus group and HC. The computing process was done with LIMMA package in R. Genes with log fold-change (logFC) greater than or equal to 1 and p-value < 0.05, were regarded as differentially expressed and selected for further functional enrichment analysis. We used the clusterProfiler package in R (Guangchuang Yu, 2011) to perform Gene Ontology (GO) and Kyoto Encyclopedia of Genes and Genomes (KEGG) enrichment analyses on DEGs, respectively. GSEA was performed on the gene expression matrix through the clusterProfiler package and “c7.immunesigdb.v7.4.entrez.gmt” was selected as the reference gene set. A false discovery rate (FDR) < 0.25 and p < 0.05 were considered as significant enrichment.

### Identification of candidate RNAs and development of an integrated mRNA–lncRNA co-expression signature

The co-expression relationship between DEGs was investigated by Pearson’s correlation measures, and modules were detected using WGCNA package in R software (Langfelder & Horvath 2008). Scale-free topology fit index was set as 0.9 as a function of the soft-thresholding power. Edges with weight > 0.1 were selected to construct the co-expression network in Cytoscape (version 3.7.0) software (Broad Institute, Inc., Massachusetts Institute of Technology, and Regents of the University of California).

### RNA fluorescence in situ hybridization (FISH)

The FISH assay was performed to detect and localize LINC01588 and NOP14-AS1 in SOMC of pemphigus patients. The probes of LINC01588 and NOP14-AS1 were synthesized by the ServiceBio Company (China) and labeled with fluorescent dye. The Servicebio ™ FISH Kit (Servicebio Company, Wuhan, China) was used to carry out RNA FISH assay according to the procedure provided by the manufacturer.

### Immunohistochemistry

Skin tissues were fixed and stained with hematoxylin. For Immunohistochemistry analysis, deparaffinized sections were washed with phosphate-buffered saline (PBS) and then treated with 3% hydrogen peroxide for 5 min. The sections were blocked with 10% normal goat serum in Tris–HCl-buffered saline or horse serum in PBS for 1 h and then incubated with primary anti-NCAM1/CD56 antibodies(Clone number: EP2567Y; Abcam, Waltham, USA) at a concentration of 1:200 for 1 h at room temperature or overnight at 4℃. After washing, the sections were incubated with appropriate secondary antibodies (biotin-conjugated IgG; Servicebio, Wuhan, China). The staining intensity was measured in three fields of every section and quantified morphometrically using Image J software.

## Results

### Identification of DEGs in both SIMC and PBMC datasets

**Fig.1:**
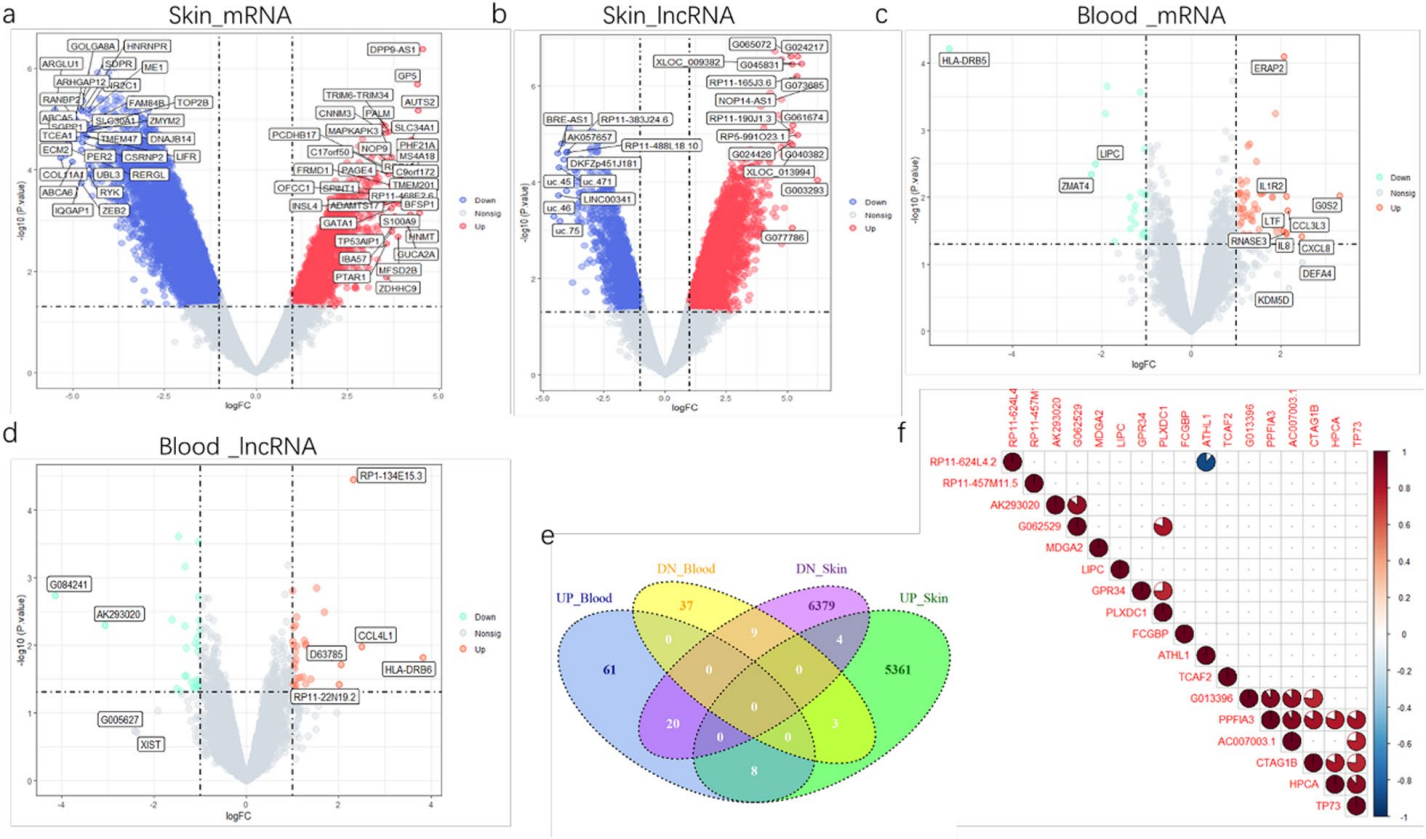
Gene expression and correlation analysis. The volcano plot illustrates DEGs (including mRNA and lncRNA) between healthy control and pemphigus after analysis of **a** mRNA of SIMC. **b** lncRNA of SIMC. **c** mRNA of PBMC.**d** lncRNA of PBMC. The y-axis indicates the statistical significance expressed as the log10 of the p-values and the x-axis shows the rate of expression change between experimental groups in log2 base (log2 Fold change), p Value cutoff = 0.05, LogFC cut 1 or – 1. **e** Authentication of 17 common DEGs in SIMC and PBMC datasets trough Venn diagrams. **f** Co-expression analysis of common DEGs in SIMC and PBMC. Pairwise correlations based on expression levels were computed for each pair of genes in the matrix shown above. Only genes with a Pearson coefficients value > 0.75 and p value < 0.05 are shown. Positive correlations are indicated in red shades and negative ones in blue shades. DEGs, differentially expressed genes. Nonsig, not statistically significant

SIMC Expression profiling data (4 patients and 4 controls, Additional file 1: Table S1) of 40,173 LncRNAs and 20,730 protein coding mRNAs were obtained by leveraging microarray analysis. PBMC expression data (4 patients and 4 controls, Additional file 2: Table S2) were obtained from our previous study [12]. After consolidation and normalization of the microarray data, DEGs were separately screened in each dataset. A total of 11,798 transcripts were differentially expressed at a significant level in SIMC while only 159 transcripts in PBMC comparing patients to control. Out of the DEGs in SIMC, there were 6829 mRNAs (1864 up-regulated and 4965 down-regulated) and 4969 lncRNAs (3515 up-regulated and 1454 down-regulated). As for DEGs of PBMC, there were 79 mRNAs (57 up-regulated and 22 down-regulated) and 60 lncRNAs (33 up-regulated and 27 down-regulated). DEGs are shown by volcano plots (Fig. 1a–d).

Next, those common DEGs in SIMC and PBMC were highlighted, as both types of cells have been proven to produce Dsg-autoantibodies, hence the common DEGs may provide more information on pemphigus-driver genes. Surprisingly, only 17 genes overlapped in two datasets (Fig. 1e), which constitute 12.23% of DEGs in PBMC dataset and only 0.14% of DEGs in SIMC dataset. Then the correlation heatmap were constructed for these 17 genes (Fig. 1f). Examining the statistically significant pair-wise correlation (p-value < 0.05 for Pearson co-efficient), one cluster of positively correlated genes were detected. The cluster included the mRNAs PPFIA3, GTAG1B, HPCA, TP73, and the lncRNAs G013396 and AC007003.1.

### Different immune cell subtype composition in peripheral blood and skin lesions

**Fig.2:**
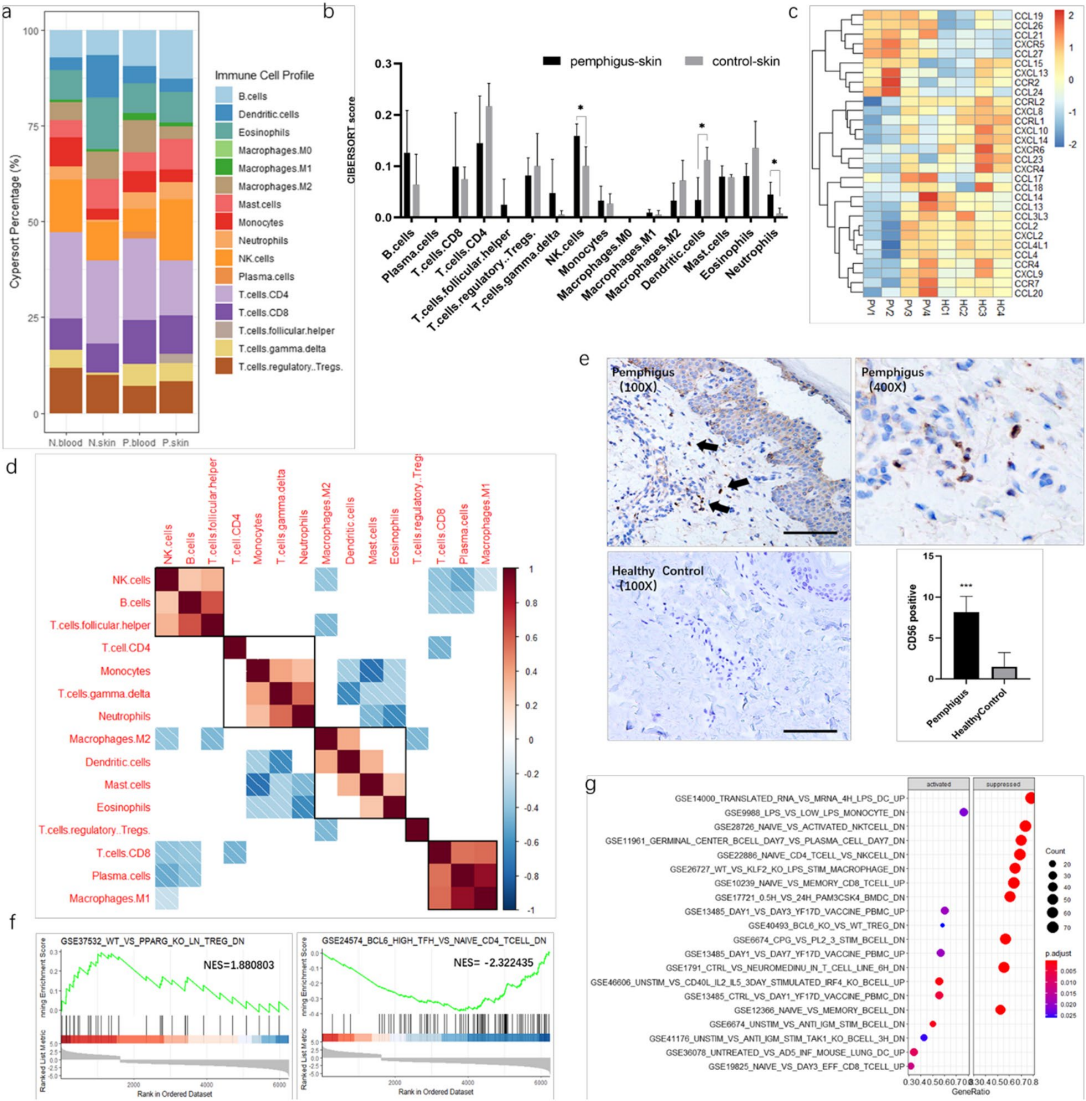
Identification and characterization of Immune cell subtypes in SIMC and PBMC. **a** the proportions of immune cells calculated by CIBERSORT, N: controls, P: pemphigus patients. **b** Barplot of CIBERSORT scores of SIMC in pemphigus and control skin samples. **c** the heatmap of deferentially expressed chemokine and chemokine receptor in SIMC between pemphigus patients and healthy controls. **d** Correlation heatmap of different types of immune cells. **e**Immunohistochemistry staining of CD56 in skin, CD56: NK cell marker. **f** GSEA enrichment plots of GSE37532, and GSE24574. Normalized enrichment score (NES) indicated the analysis results across gene sets. **g** GSEA analysis result of SIMC database

We expected the differences of DEGs between the SIMC and PBMC mainly stem from the immune cell subtype composition. To better understand and characterize the differences between SIMC and PBMC, we used the CIBERSORT deconvolution algorithm to identify immune cell infiltration characteristics in pemphigus patients. SIMC from pemphigus patients had a higher NK cells, and neutrophils infiltrating level, compared with control, while PBMC showed no such a difference (Fig. 2a, b, Additional file 3: Fig. S4). An increased number of infiltrating NK cells in pemphigus lesions was confirmed by immunohistochemistry. Dermal layer of pemphigus lesions had significantly more CD56 positive cells compared with normal skin (Fig. 2e).

To further study the immune environment, we generated the chemokines and chemokines receptors based on the global gene expression analysis (Fig. 2c). Notably, CCL19, CCL26, CCL27 and CXCR5 were highly expressed chemokines and chemokine receptors detected in pemphigus group (Fig. 2c). The correlation heatmap of the 16 types of immune cells revealed that B cell abundance had a positive correlation with Tfh cell and NK cell abundances (p < 0.05). Moreover, plasma cell (PC) abundance had a positive correlation with type I macrophage (M1) abundance, and dendritic cell population was positively correlated with type II macrophage (M2) abundance (Fig. 2d).

To further investigate the immunological mechanisms involved in pemphigus lesions, the GESA package in R was used to analyze the SIMC global gene expression data. The results displayed that the related genes were significantly enriched in immune cell activation processes and immune cell differentiation signatures (Fig. 2f). Among top scoring activated gene sets, from GSEA database (https://www.gsea-msigdb.org/gsea/index.jsp), immune cell differentiation signatures, GSE24574 (related to BL6^hi^ Tfh cells) and GSE37532 (related to regulatory T cell’s inability for maturation) were shown (Fig. 2g).

### GO and KEGG analysis for the DEGs in SIMC and PBMC datasets

**Fig.3:**
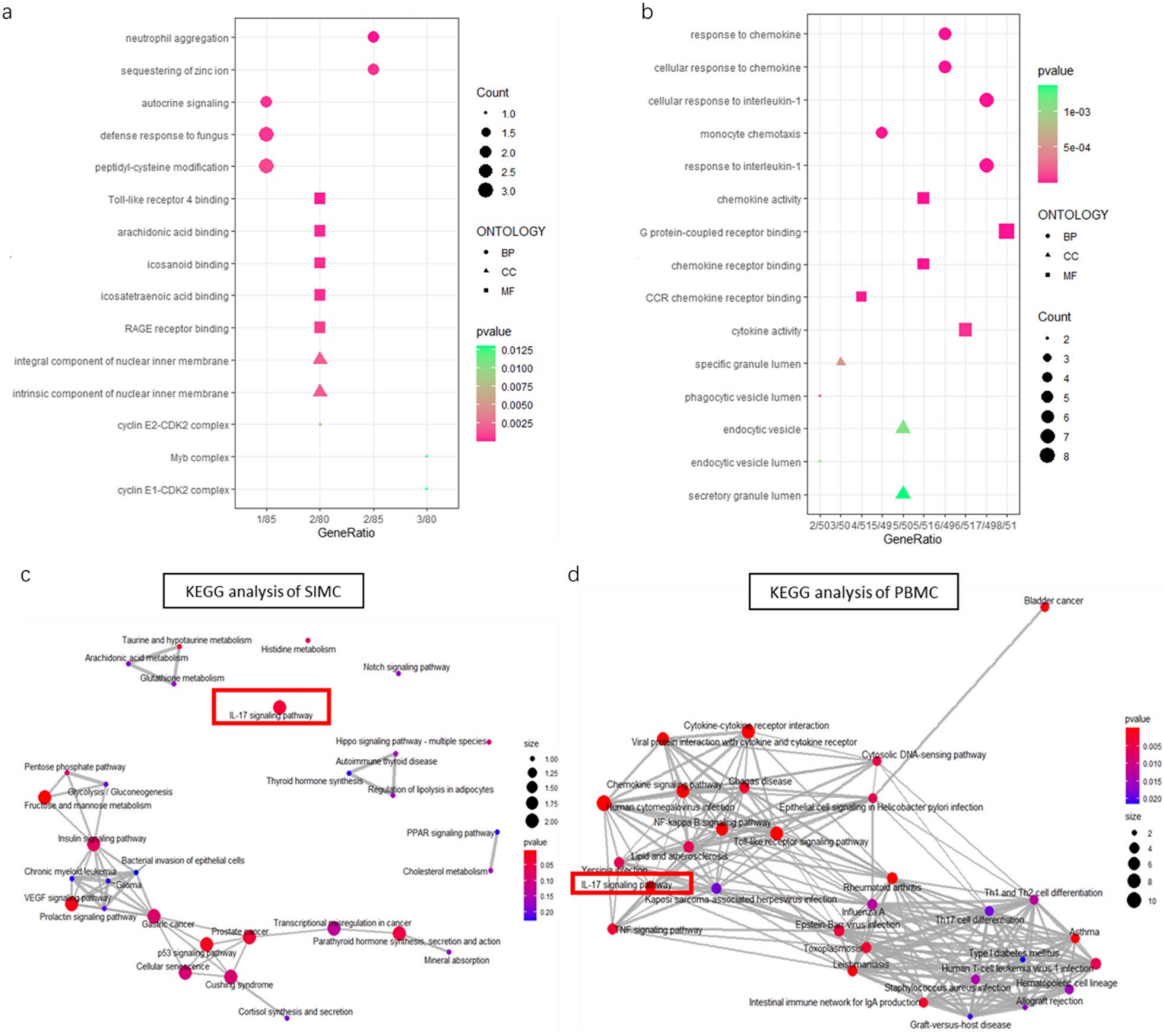
GO and KEGG pathway enrichment analysis of DEGS. Top 15 GO term enrichment results of top 100 DEGs of **a** SIMC. **b** PBMC. Purple bars show molecular function, orange bars show cellular component and green bars show biological process. Vertical axis represents the number under the GO term. Enrichment Map for top30 KEGG enrichment results of **c** SIMC. **d** PBMC

To better illustrate the unique roles of SIMC, we performed functional enrichment analyses on SIMC and PBMC datasets, respectively. The GO analysis results showed that DEGs in both datasets were mostly enriched in biological process terms. The up-regulated genes in SIMC were mainly enriched in neutrophil aggregation, defense response to fungus, Toll-like receptor 4 binding and RAGE receptor binding, among others (Fig. 3a). On the other hand, in PBMC group, over-represented genes were enriched in immunological processes including cellular response to interleukin-1, response to chemokines, CCR chemokine receptor binding, phagocytic vesicle lumen as well as endocytic vesicle, among others (Fig. 3b). KEGG pathway analysis showed the related genes in PBMC groups, were involved in viral infection related pathways, Chemokine signaling pathway, Cytokine-cytokine receptor interaction, and interleukin-17 (IL-17) signaling pathway (Fig. 3d). In SIMC group, most enriched KEGG terms were fructose and mannose metabolism, VEGF signaling pathway, p53 signaling pathway, PPAR signaling pathway, and IL-17 signaling pathway among others (Fig. 3c).

### Identification of LINC01588 as a potential epigenetic regulator in Treg/Th17 balance

**Fig. 4:**
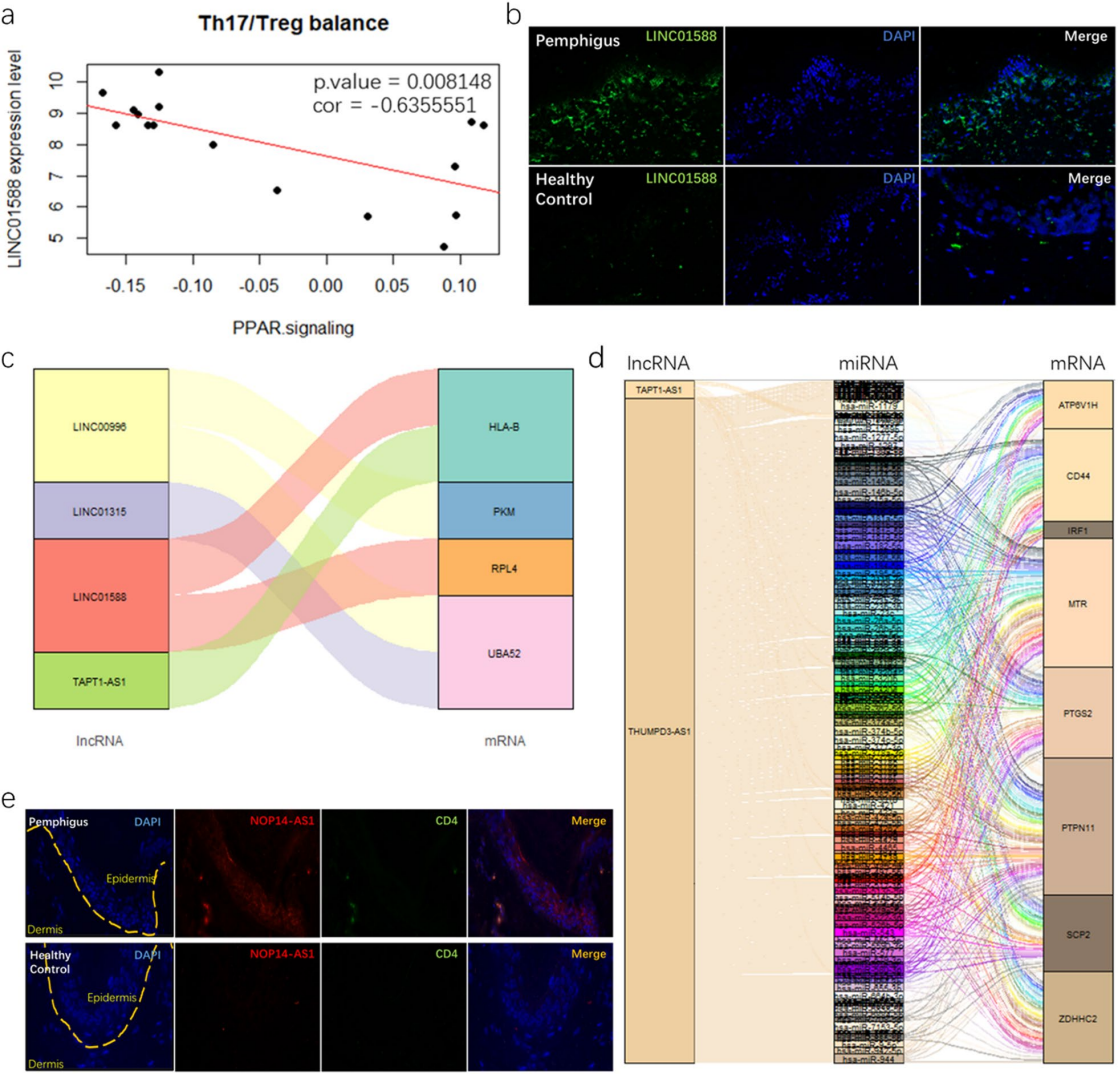
**a** correlation analysis between LINC01588 expression and PPAR signaling way. **b** FISH staining patterns of LINC01588 in pemphigus lesions and normal skin. **c** Sankey diagram for immune associated lncRNAs and mRNAs. **d** ceRNA network analysis of lncRNAs, miRNAs and mRNAs, each rectangle represents a gene and connection degree of each gene is visualized based on the size if the rectangle. **e** FISH colocalization of NOP14-AS1 and CD4 in pemphigus lesions and normal skin

LncRNAs has emerged as critical regulators in the immune system. To explore its contribution to the immune phenotype of SIMC and PBMC, we looked at the expression level of immune cell specific lncRNAs. 22 immune-cell specific lncRNAs were differentially expressed in skin lesions. These lncRNAs are related to dendritic cells, CD8+ T cells, T helper 17 cells and NK cells, among others. While in PBMC, only TAPT1-AS1, which is specific to activated B cell was found (Table 1). It is well established that pemphigus is an IL-17 related immune response [13–16]. Therefore, we next focused on Th17 cell specific LncRNA LINC01588. The PPAR signaling pathway score was calculated by averaging the expression values of genes in the PPAR signaling pathway. The PPAR signaling pathway gene-set was acquired from the Nanostring platform. The result showed that LINC01588 had a significant negative correlation with the PPAR signaling pathway (p-value < 0.01) (Fig. 4a). FISH results showed that LINC01588 had a higher expression in pemphigus lesions (Fig. 4b). Higher expression of NOP14-AS1, one of most up-regulated lncRNA in pemphigus lesions, was also confirmed using FISH. Furthermore, NOP14-AS1 was co-expressed with CD4 (Fig. 4e). To explore the relationship between lncRNA and mRNA, we next constructed a lncRNA-mRNA and lncRNA-miRNA-mRNA networks (Fig. 4c, d), based on the data from ENCORI database [17, 18].

**Table 1:** Differentially expressed immune associated lncRNAs in SIMC

SYMBOL	ID	Immune cell type	logFC	AveExpr	p value
GS1-124K5.4	ENSG00000237310	CD8 T cell resting	− 2.55936	4.132913	5.26E-05
A1BG-AS1	ENSG00000268895	Plasmacytoid dendritic cells	2.229881	5.300229	0.000375
LINC01588	ENSG00000214900	T helper 17	2.466711	6.913147	0.00041
LOC101927811	LOC101927811	Dendritic cells activated	2.387872	7.123568	0.000481
DBH-AS1	ENSG00000225756	Myeloid dendritic cells	2.150917	5.761338	0.000642
THUMPD3-AS1	ENSG00000206573	Dendritic cells activated	− 2.03061	3.671935	0.000729
LOC103611081	ENSG00000255455	CD4 T cell resting	− 1.96291	6.592654	0.001649
LINC00996	ENSG00000242258	Plasmacytoid dendritic cells	2.992853	9.091972	0.001662
LINC00597		NKT activated	− 3.30812	5.175846	0.00206
TAPT1-AS1	ENSG00000263327	B cell activated	− 1.82827	4.256622	0.003663
HOXB-AS1	ENSG00000230148	Mast cells activated	1.651809	4.861386	0.004255
LINC01315	ENSG00000229891	Dendritic cells resting	− 2.05771	6.08522	0.004324
LINC01296		Mast cells activated	− 1.99584	4.991194	0.004599
LINC01234	ENSG00000249550	Mast cells activated	2.820071	4.198001	0.00588
LOC100996455	ENSG00000181908	Plasmacytoid dendritic cells	2.028425	5.02938	0.005897
LOC100506990		CD8 T cell activated	− 1.65011	4.371577	0.010919
CYB561D2	ENSG00000271858	Mast cells activated	2.17753	7.523808	0.017323
LINC00852	ENSG00000231177	Plasmacytoid dendritic cells	− 1.26985	3.66593	0.01772
LINC00668	ENSG00000265933	CD8 T cell resting	1.451025	3.953123	0.019095
NAV2-AS4	ENSG00000254622	CD8 T cell resting	1.159277	4.208695	0.026467
LOC100130872	LOC100130872	NK resting	1.727115	9.421826	0.036093
LOC643072	LOC643072	Neutrophils	1.020082	4.22196	0.044587

### WGCNA network module mining reveal pemphigus associated pattern

**Fig. 5:**
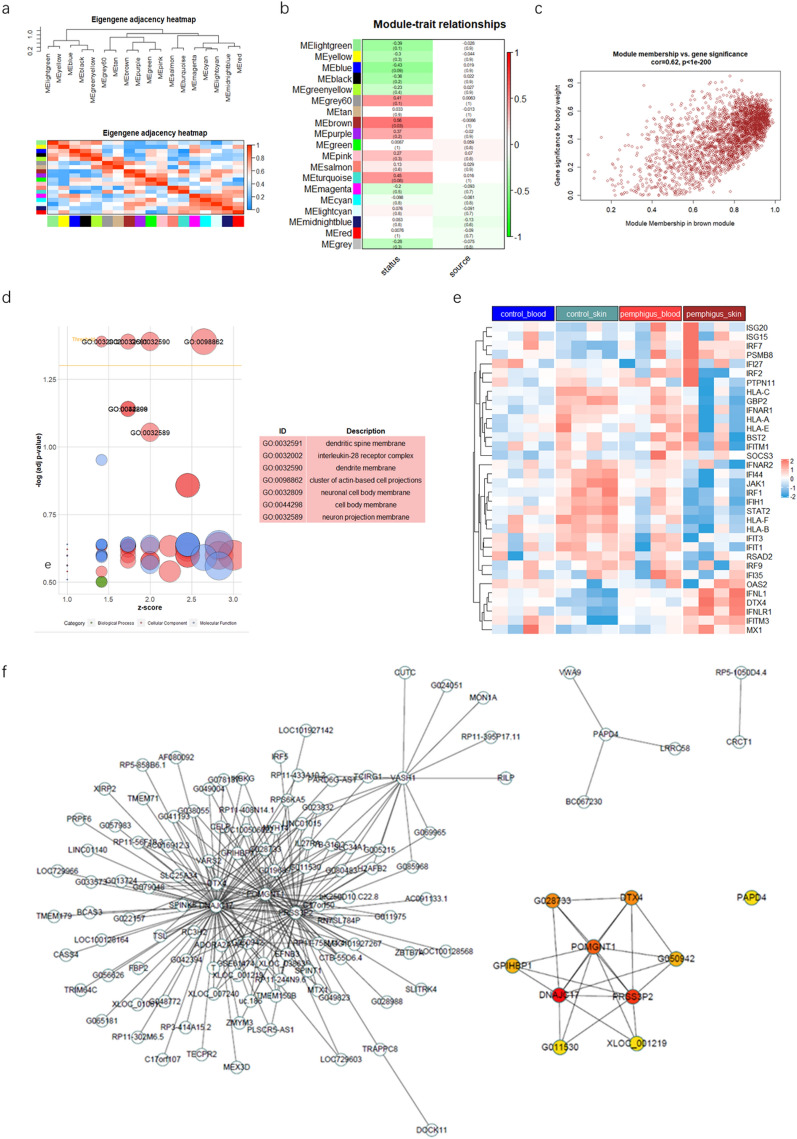
Identification and characterization of pemphigus-associated modules using WGCNA. **a** Eigengene adjacency heatmap shows correlations between modules. **b** The heatmap to show the correlation between module eigengenes and disease status or sample source relationship of 19 modules. P-value is shown in brackets. Status: disease status. Source: sample source. **c** the scatter plot of module eigengenes in brown module. **d** GO enrichment result of brown module genes. **e** heatmap of genes associated to type I IFN signaling pathway.**f** the protein–protein interaction network of genes in brown module. Ten hub genes identified by the ‘cytoHubba’ plugin via mixed character calculation. The significance of hub genes increases with increasing intensity of the dot color

The microarray dataset contains over 20 thousand gene expression data. Merely focusing on DEGs may lead to overlooking potentially significant results. Therefore, we next integrated the expression matrices of all 16 samples in both SIMC and PBMC datasets and identified pemphigus-related preserved gene modules in two datasets using weighted gene co-expression network analysis (WGCNA). After batch effect removal (Additional file 3: Fig. S1), scale free topology criterion was applied as follows: the soft threshold power of β was 10 when scale-free topology model fit R^2^ was maximized at 0.85 (Additional file 3: Fig. S2). A total of 19 modules were identified in the network. The parameters were set as follow: a relatively large minimum module size (size = 30), a medium module detection sensitivity (deepSplit = 2), and the cut height for merging modules (height = 0.25). The modules whose eigengenes were correlated above 0.75 were merged. Dendrogram clusters and heatmaps are shown in the attachment (Additional file 3: Fig. S3). The heatmaps of eigengene adjacency and module-trait relationships showed that, in the 19 modules, the brown module was positively correlated with occurrence of pemphigus. None of the modules correlated with sample sources were found (Fig. 5a, b).

A multi-dimensional scaling plot was generated to evaluate the expression of genes in the brown module (Fig. 5c). The brown module includes 1462 mRNAs and 1129 lncRNAs. To screen out the hub genes, we calculated the intramodular connectivity of all genes in the module. According to the multi-dimensional scaling plot, we defined genes of high gene significance (GS > 0.5) and high intramodular connectivity (IC > 0.8) as main contributor genes in the brown module. Then, functional enrichment analysis was used to investigate the module function. The bubble plot illustrates that main contributor genes were markedly enriched in dendritic spine membrane, interleukin-28 receptor complex, dendrite membrane, cluster of actin-based cell projections, neuronal cell body membrane, cell body membrane and neuron projection membrane (adjust p value < 0.01, Fig. 5d). We also constructed the Protein–Protein Interaction Networks (PPIs), to explore molecule interaction in the brown module on protein level. PPIs of these genes were constituted by STRING and visualized by Cytoscape. The genes with combined scores greater than 0.4 were selected for constructing networks. The ten hub genes were chosen using CytoHubba plugin and were as follows: DNAJC17, PRSS3P2, POMGNT1, DTX4, G028733, GPHBP1, G011530, XLOC_001219, PAPD4, and G050942 (Fig. 5f). In the enrichment results, genes such as IFNL, IFNLR (Additional file 4: Table S3) and hub gene DTX4 were type I IFN related. Type I IFN signature has been reported in many autoimmune diseases, such as systemic lupus erythematosus. Hence, we displayed type I IFN signaling pathways associated genes in a heatmap manner. However, most genes were not up-regulated in pemphigus (Fig. 5e). The type I IFN signaling pathways gene-set used above was generated using the Nanostring panel database (https://www.nanostring.com/products/ncounter-assays-panels/panel-selection-tool/).

## Discussion

The main characteristic of pemphigus is autoantibodies targeting Dsg1 and Dag3. Current studies regarding pemphigus have largely focused on PBMC. Our group has previously reported that local immune response in pemphigus lesions may play an important role in pemphigus pathogenesis. Yet, an advanced understanding of the altered biological pathways and molecular mechanisms in SIMC is needed to illustrate its role.

Mounting evidence indicates that skin-resident immune cells play an important role in maintaining skin immunity homeostasis [19]. Crosstalk between innate immune cells and adaptive immune cells has become a research hotspot. They co-operate to achieve finely balanced state of the immune system that maintains tolerance to self-antigens. To explore this aspect, we illustrated the immune landscape of pemphigus blood and skin by applying CIBERSORT, a computational approach for inferring leukocyte and lymphocyte representation in bulk transcriptomes. The results showed that pemphigus lesions had a higher neutrophil infiltrating level which is in line with the GO enrichment results (Fig. 3a). By investigating the correlation between different cell types, we found that M1 abundance correlated with PC abundance. Xu et al. [20] Identified macrophages as important players in the induction of PC terminal differentiation through the secretion of CXCL10. Our team has reported CD138 + PCs in pemphigus lesions and confirmed they were able to secrete Dsg-specific antibodies via in vitro experiments [5]. These findings indicated that M1 might be a potential catalyst in pathological progression and should be the highlight of further studies.

In pemphigus lesions, we found a significantly increased infiltration of NK cells and its abundance was positively correlated with B cell abundance. NK cells have been traditionally considered as innate immune cells, but recently they have been proven to be mediating adaptive immunity and have vaccination-dependent, antigen-specific, and long-lived immunological memory characteristics [21]. NK cells exhibit immunoregulatory function in the pathogenesis of myasthenia gravis (MG) [22]. The killing effects of NK cells on CD4 + T cells and Tfh cells were impaired in MG patients, resulting in promotion of the differentiation and activation of Tfh cells. The role of pemphigus lesion infiltrating NK cells needs further elucidation. Development of a bispecific antibody therapy may be worth pursuing. Bispecific antibodies are monoclonal antibodies that targets two different epitopes [23]. One end binds to target cells like tumor cells, in this case autoantibody producing B cells. Another end binds to killing cells like T cells or NK cells. Even though Rituximab (RTX) therapy have been tested effective, patients with a high baseline frequency of memory class-switch IgG B-cells (25% among DSG-3 specific B-cells) still had active disease after RTX treatment [24]. Meanwhile, patients also face risk of severe infection due to immunosuppression. An alternative treatment like bispecific antibody that activates local NK cell to kill Dsg-specific Ig + B cells will be promising.

Chemokines and its receptors could be a major contribution for the enriched infiltration of immune cells in pemphigus lesions. In this study, the global gene expression analysis displayed that the most highly expressed chemokine is CCL27, and chemokine receptor is CXCR5. CCL27 (CTACK) is an inflammatory chemokine which binds to CCR10 and is associated with homing of memory T cells to sites of inflammation. Bernhard etc. established the pivotal role of CCL27-CCR10 interactions in T cell-mediated skin inflammation using mice models. Their data showed that, lymphocytes accumulate at sites of CCL27 injection and neutralization of CCL27-CCR10 interaction by administration of anti-CCL27 neutralizing antibodies can impair lymphocyte recruitment [25]. The accumulated body of evidence indicates that skin-associated immuno-surveillance may be influenced by the CCL27/CCR10 interaction. Yet, its role in pemphigus remains elusive. CXCR5 is mainly expressed on the cell surface of B cells and Tfh cells. Our previous study described the formation of ELSs in pemphigus lesions. It is a structure constituted by T cell and B cells, serving as a local factory for autoantibody production. In the previous study, we detected the mRNA expression level of selective chemokines. CCL5 and CCL20 were found to be highly expressed in pemphigus lesions [5]. At this stage of understanding, we believe many chemokines and their receptors are involved in the enrichment of immune cell in pemphigus lesion. Nevertheless, it is important to note, that the present evidence relies on mostly transcriptomic data. More experiments at protein level need to be conducted in order to complete the overall picture of skin homing factors in pemphigus.

We also compared the functional analysis results of SIMC with that of PBMC to better understand the unique roles of SIMC. The GO analysis results showed that PBMC had an over-representation of inflammatory cytokines and chemokines, while SIMC had a signature of neutrophil aggregations and other metabolism-related pathways. These results indicated that SIMC and PBMC had vastly different functional phenotypes. Interestingly, SIMC and PBMC shared similarity in the KEGG results. The IL-17 signaling pathway was over-represented in both SIMC and PBMC which is consistent with previous reports [13–16]. Increasing evidence has shown that Dsg1/3-specific autoantibody production may be promoted by IL-17 + T cells. Cellular response to IL-1 term was enriched in PBMC. IL-1 is a strong inducer of innate IL-17 who in turn, recruits IL-1-secreting myeloid cells [26], suggesting that a positive feedback cycle may exist in pemphigus. Holstein et al. [13] has shown that neutrophil aggregation was the most significantly enriched GO term in pemphigus skin lesions which was also confirmed by the GO analysis of our study. Neutrophil was reported to also produce IL-17 [27–29]. However, whether the neutrophil aggregation contributed to local IL-17 production needs further investigation.

Many differentially expressed lncRNAs were also screened out using bioinformatics techniques. Recent evidence has shown that lncRNAs are expressed in a highly lineage-specific manner and control the differentiation and function of both innate and adaptive cell types [30]. The CIBERSORT and GSEA results demonstrated that SIMC had distinct immune cell subtype composition and immunophenotypes. We suspected that lncRNAs are likely to function as epigenetic regulators in SIMC and contributed to these differences. For this reason, we examined immune cell specific lncRNAs expression level and constructed a ceRNA network. Immune cell-specific lncRNAs were defined by Zhou et al. [31]. Twenty-three immune-cell-specific lncRNAs were found differentially expressed in pemphigus SIMC compared with healthy control. Our correlation results showed that a Th17 specific lncRNA, LINC01588 expression level was negatively correlated with the PPAR score meaning that LINC01588 may be a negative regulator of the PPAR signaling pathway which is required for Treg cells maturation.

We attempted to build a mRNA and lncRNA expression network using WGCNA to identify a disease associated gene signature. Out of 19 modules, only the brown module was related to disease status. The main contributor genes (GS > 0.5 & IC > 0.8) in the module were further analyzed. These genes were functionally enriched by GO terms and ten hub-genes screened out using CytoHubba based on their protein–protein interaction. Because type I IFN related genes were enriched and It was well established that type I IFN response is highly correlated with autoimmune diseases such as cutaneous lupus [32]. We deduced that type I IFN signaling pathway may play a role in pemphigus disease. However, the heatmap showed that most genes related to type I IFN signaling pathway were not up-regulated in SIMC, suggesting type I IFN signaling probably is not the key pathogenic mechanism.

In this study, we sought to further explore the role of immune cell infiltration in pemphigus and identify novel genes in its pathogenesis. However, there are some limitations to our study. Firstly, this study had a relatively small sample size given the fact that pemphigus is a rare disease. And for the same reason, only limited laboratory experiments were conducted to validate these results. Secondly, the exact mechanism of interaction between immune cells and immune reaction regulated by lncRNA needs to be further investigated. Lastly, the bioinformatic analyses were based on limited transcriptomic data. Therefore, our findings still need verification through in vitro and in vivo experiments.

## Conclusion

Overall, the present study represents the first transcriptional profiling of SIMC. Our study is important in the context of a prior report that illustrated the unique gene expression pattern and immune landscape in pemphigus lesions. We showed the crosstalk between innate and adaptive immune cells, like macrophages and plasma cells. Our study is also the first to demonstrate an increased infiltration of NK cell in pemphigus lesions. In addition, we found that LINC10588 was negatively correlated to PPAR signaling which may be related to the pathogenesis of pemphigus.

## Supplementary Information

**Additional file 1.** Supplementary figures

**Additional file 2.** SIMC expression profiling data

**Additional file 3.** PBMC expression profiling data

**Additional file 4.** Enrichment results of the brown module

## Data Availability

The expression datasets of PBMC and SIMC are attached as additional files.

## Abbreviations

ELSs: Ectopic lymphoid-like structures
Dsg: Desmoglein
SIMC: Skin lesion infiltrating mononuclear cells
PBMC: Peripheral blood mononuclear cells
CIBERSORT: Cell-type identification with relative subsets of known RNA transcripts
NK cells: Naïve natural killer cells
M1: Type 1 macrophages
Th17: T helper 17 cells
Treg: Regulatory T cells
ncRNA: Non-coding RNAs
ceRNA: Competing endogenous RNA
FISH: RNA Fluorescence in Situ Hybridization
NES: Normalized enrichment score
WGCNA: Weighted gene co-expression network
GS: Gene significance
IC: Intramodular connectivity
GO: Gene Ontology
KEGG: Kyoto Encyclopedia of Genes and Genomes
