# Supplementary material for: Transcriptomic profiling of pemphigus lesion infiltrating mononuclear cells reveals a distinct local immune microenvironment and novel lncRNA regulators
Source: J Transl Med. 2022 Apr 21;20:182. doi: 10.1186/s12967-022-03387-7 (PMC9027862; doi:10.1186/s12967-022-03387-7)
Supplement: Supplementary file 3 — Additional file 3. PBMC expression profiling data [file 12967_2022_3387_MOESM3_ESM.docx]

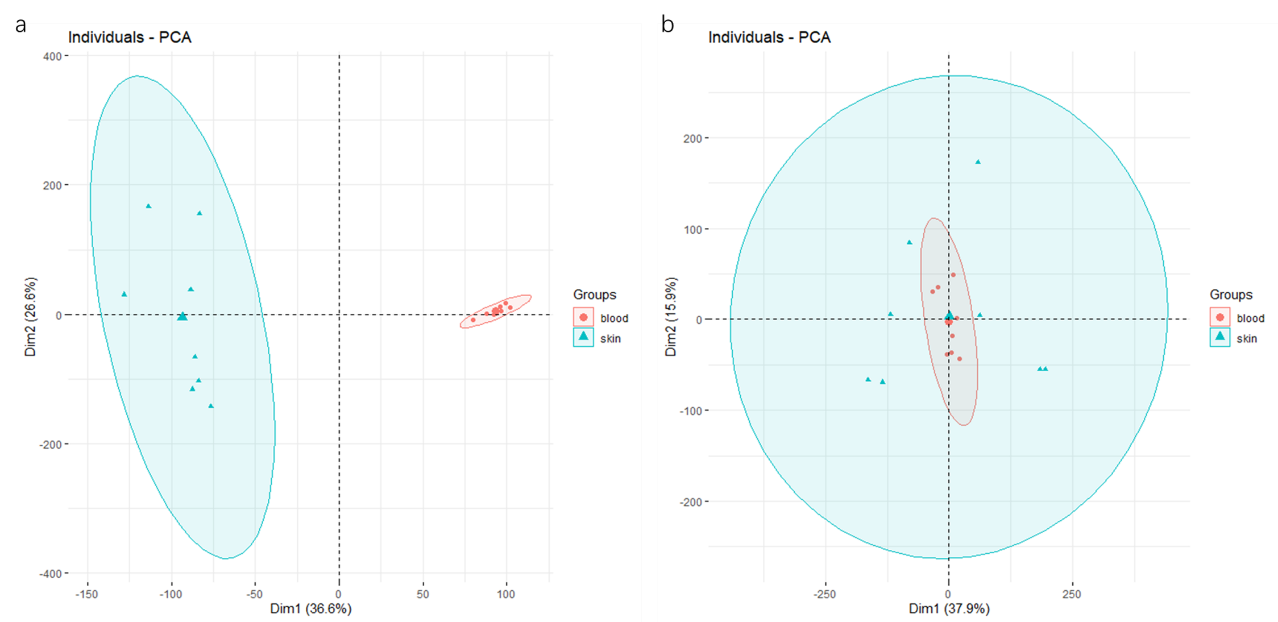


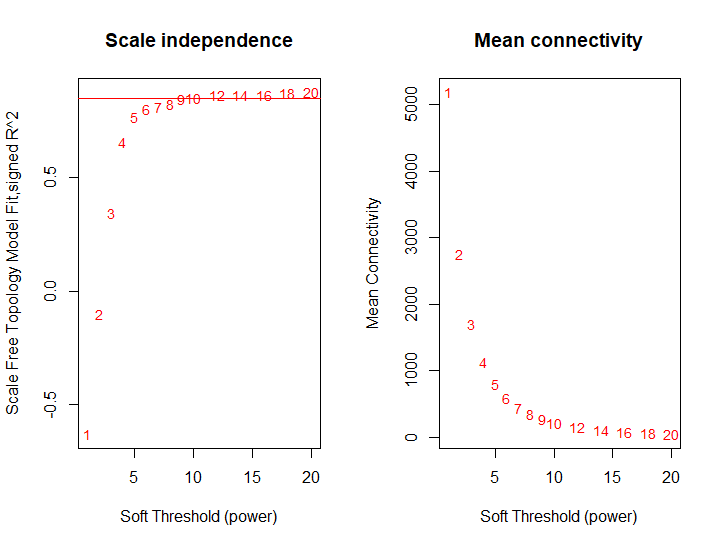
Fig s1 Removal of batch effect between PBMC and SIMC **a** before **b** after


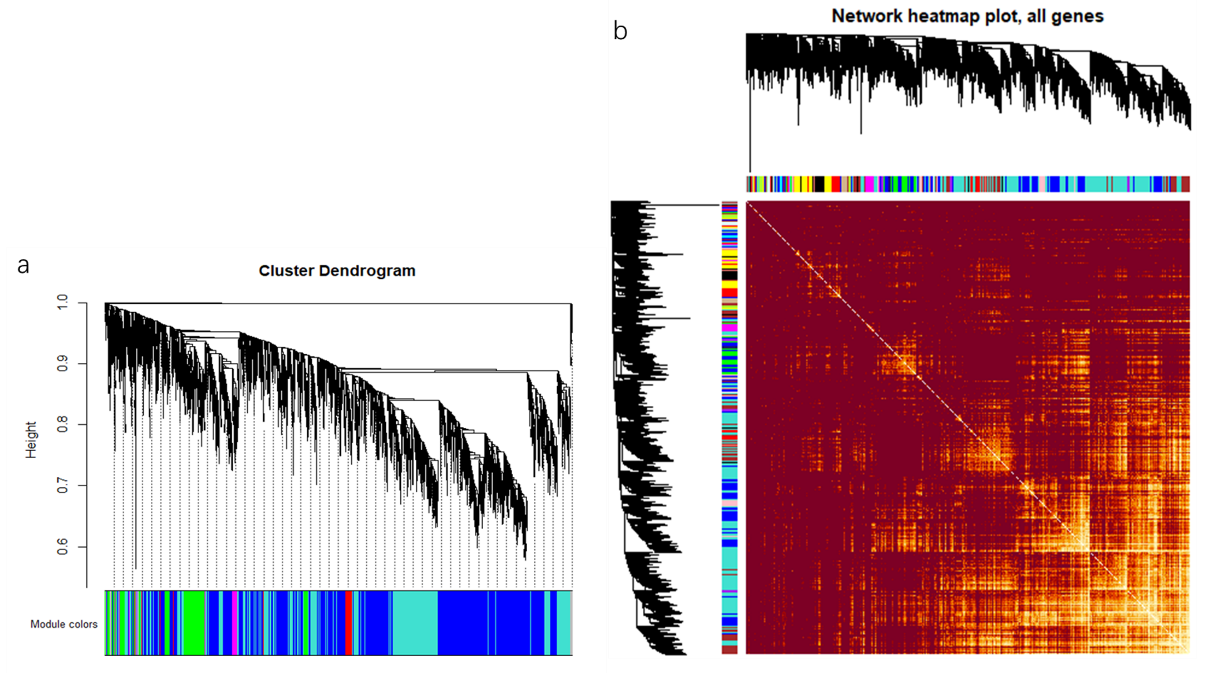
Fig s2 Scale free topology criterion

Fig s3 Dendrogram **a** clusters and **b** heatmaps


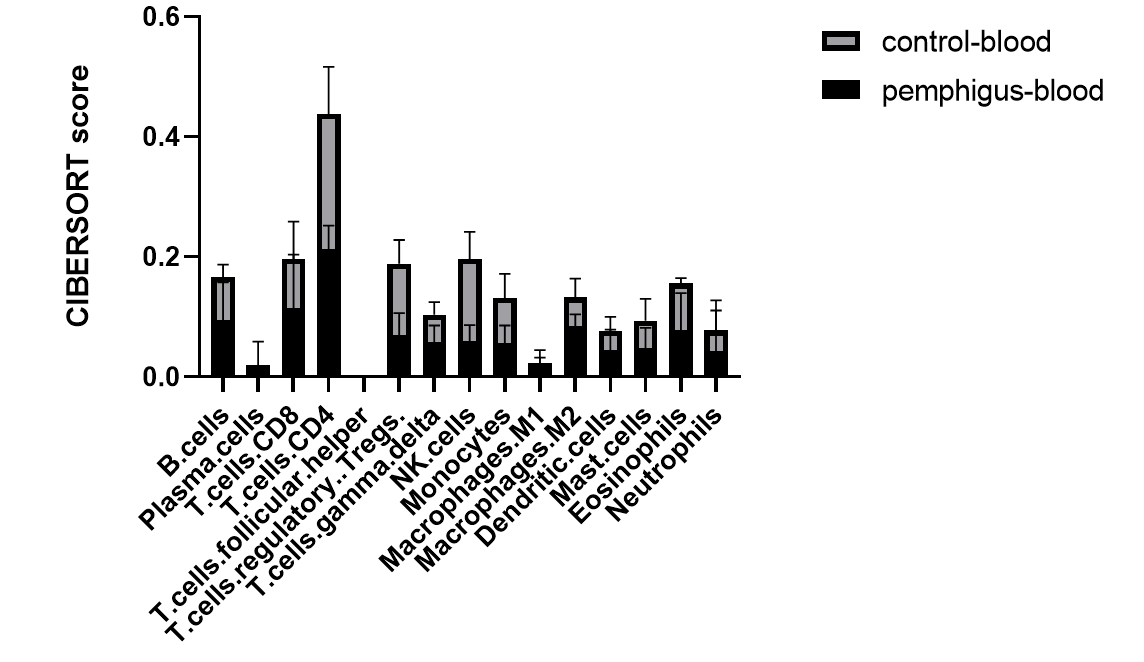


Fig s4 CIBERSORT results of PBMC
